# Cardiologists’ Diagnostic and Therapeutic Approaches in the Outpatient Management of Patients with Chronic Coronary Syndrome in Turkey: An Observational–Registration Study

**DOI:** 10.3390/jcdd13090455

**Published:** 2026-09-10

**Authors:** Abdulla Arslan, Oyku Gulmez Ozkaya, Fatih Aytemiz, Murat Küçükukur, Emre Asiltürk, Ufuk Iyigün, Muhammed Bayram, Halil Fedai, Şahbender Koç, Duygu Malkoç, Mustafa Doğduş, Ömer Kümet, Murat Bayrak, Önder Öztürk, Ajar Koçak, Sefa Erdi Ömür, Lütfü Bekar, Özkan Kayhan, Fahrettin Katkat, Cansu Öztürk, Emrah Yeşil, Alkame Akgümüş, Ahmet Balun, Aslan Erdoğan, Ayşegül Ülgen Kunak, Kenan Toprak, Fahri Er, Ebru Serin, Serhat Çalışkan, Sinan İnci, Mehdi Zoghi

**Affiliations:** 1 Cardiology Department, Başkent University, Istanbul 34000, Türkiye; gulmezoyku@yahoo.com; 2 Department of Cardiology, City Hospital of Manisa, Manisa 45030, Türkiye; fatihaytemiz83@gmail.com; 3 Department of Cardiology, İzmir City Hospital, İzmir 35530, Türkiye; drmuratkucuk055@gmail.com; 4 Department of Cardiology, Antalya Training and Research Hospital, Antalya 07100, Türkiye; e-asilturk@hotmail.com (E.A.); dr.ulgen@hotmail.com (A.Ü.K.); 5 Akdeniz Health and Life Foundation, Department of Cardiology, ASV Yaşam Hospital, Antalya 07100, Türkiye; druiyigun@hotmail.com; 6 Cardiology, Sancaktepe Şehit Prof. Dr. İlhan Varank Eğitim ve Araştırma Hastanesi, Sancaktepe 34785, Türkiye; muhammedbyrm@gmail.com; 7 Department of Cardiology, Faculty of Medicine, Harran University, Şanlıurfa 63190, Türkiye; drhalilfedai@gmail.com (H.F.); kentoprak@hotmail.com (K.T.); 8 Department of Cardiology, Ankara Sanatoryum Training and Research Hospital, Ankara 06290, Türkiye; sahbenderkoc@hotmail.com; 9 Department of Cardiology, Faculty of Medicine, Ege University, İzmir 35040, Türkiye; malkocduygu@hotmail.com (D.M.); mehdi_zoghi@hotmail.com (M.Z.); 10 Department of Cardiology, Faculty of Medicine, Medical Point Hospital, İzmir University of Economics, İzmir 35330, Türkiye; mdogdus@hotmail.com; 11 Department of Cardiology, Van Training and Research Hospital, University of Health Sciences, Van 65100, Türkiye; omerkumet@hotmail.com; 12 Department of Cardiology, Antalya Kepez State Hospital, Antalya 07320, Türkiye; drmurat87@hotmail.com (M.B.); ozkan_kayhan@outlook.com (Ö.K.); 13 Department of Cardiology, Saglik Bilimleri University Diyarbakir Gazi Yasargil Training and Research Hospital, Diyarbakir 21070, Türkiye; prof.dr.onderozturk@gmail.com (Ö.Ö.); dr.cansu.ozturk@gmail.com (C.Ö.); 14 Department of Cardiology, Faculty of Medicine, Ufuk University, Ankara 06836, Türkiye; kocakajar@gmail.com; 15 Department of Cardiology, Faculty of Medicine, Tokat Gazi Osman Paşa University, Tokat 60250, Türkiye; sefaerdi61@gmail.com; 16 Department of Cardiology, Faculty of Medicine, Hitit University, Çorum 19030, Türkiye; drlutfubekar@gmail.com; 17 Department of Cardiology, İstanbul Training and Research Hospital, İstanbul 34098, Türkiye; fahrettin_katkat@hotmail.com; 18 Department of Cardiology, Faculty of Medicine, Mersin University, Mersin 33110, Türkiye; emrhyesil@gmail.com; 19 Department of Cardiology, Faculty of Medicine, Bandırma 17 Eylül University, Balıkesir 10250, Türkiye; aakgumus@bandirma.edu.tr (A.A.); ahmetbalun@gmail.com (A.B.); 20 Department of Cardiology, Cam and Sakura Training and Research Hospital, Istanbul 34480, Türkiye; aslanerdogan2011@hotmail.com; 21 Department of Cardiology, Ağrı Training and Research Hospital, Ağrı 04100, Türkiye; fahrier147@gmail.com; 22 Department of Cardiology, Sisli Hamidiye Etfal Training and Research Hospital, Istanbul 34418, Türkiye; serin.ebru@hotmail.com; 23 Department of Cardiology, İstanbul Bahçelievler State Hospital, İstanbul 34186, Türkiye; drserhat07@hotmail.com; 24 Department of Cardiology, Aksaray University School of Medicine, Aksaray 68100, Türkiye; doktorsinaninci@gmail.com

**Keywords:** chronic coronary syndrome, angina, beta-blocker, ranolazine

## Abstract

Background: Chronic coronary syndrome (CCS) remains a major cause of morbidity and mortality worldwide, and real-world data on outpatient management are limited. Methods: In total, 697 patients with previously diagnosed CCS who visited outpatient cardiology clinics at 25 centers in Turkey between June 2023 and December 2023 were prospectively enrolled and followed up for 12 weeks. Demographic and clinical characteristics, diagnostic and therapeutic strategies, a modified questionnaire derived from the Seattle Angina Questionnaire (modified SAQ-based questionnaire), and clinical outcomes were recorded. Results: The most common diagnostic modalities were exercise stress testing (31.6%) and coronary angiography (38.2%). Medical therapy (58.5%) was preferred over interventional treatment (41.5%). Among patients who were not receiving the respective medication at baseline, beta-blockers (61.8%), statins (46.5%), and ranolazine (40.4%) were the most commonly newly initiated medications. Patients in the intervention group were younger, were more frequently current smokers, had greater nitrate use, and had lower ejection fraction than those in the medical treatment group. Patient-reported responses to the modified SAQ-based questionnaire shifted toward less functional limitation, lower angina burden, and better perceived quality of life over the 12-week follow-up. Conclusions: Medical therapy was the predominant management strategy for CCS in Turkey. The observed diagnostic and therapeutic patterns reflect real-world management of patients with established CCS, with substantial use of invasive coronary angiography and high baseline utilization of guideline-directed medical therapies.

## 1. Introduction

Chronic coronary syndrome (CCS) encompasses a broad spectrum of clinical presentations of coronary artery disease during stable periods, including patients with angina or myocardial ischemia, asymptomatic coronary artery disease, and patients in the non-acute phase following acute coronary syndrome or coronary revascularization [1]. Ischemic heart disease remains a leading cause of morbidity and mortality worldwide and imposes a substantial burden on health care systems [2]. Ischemic heart disease also represents a substantial public health burden in Turkey, underscoring the importance of optimizing diagnostic and therapeutic strategies within the national health care setting [3].

The European Society of Cardiology (ESC) and the American College of Cardiology/American Heart Association (ACC/AHA) have developed comprehensive guidelines for the diagnosis and management of chronic coronary syndromes and chronic coronary disease, respectively [1,4]. These guidelines recommend individualized treatment approaches based on disease severity, clinical presentation, comorbidities, and ischemic risk. The primary objectives of CCS treatment include symptom relief, reduced frequency and severity of angina, quality-of-life improvement, and prevention of major adverse cardiovascular events. Medical therapy remains the cornerstone of CCS management. Beta-blockers and calcium-channel blockers are recommended as first-line antianginal agents for the relief of angina symptoms [4]. Angiotensin-converting enzyme inhibitors (ACEis) or angiotensin receptor blockers (ARBs) are recommended in selected patients with CCS, particularly those with hypertension, diabetes, chronic kidney disease, or left ventricular systolic dysfunction, to reduce cardiovascular events [4]. Statins remain a first-line lipid-lowering therapy for reducing low-density lipoprotein cholesterol levels and cardiovascular risk [1,4]. Antiplatelet therapy, particularly aspirin or clopidogrel according to the clinical setting, plays a central role in the secondary prevention of thrombotic events [1,4]. Revascularization strategies, including percutaneous coronary intervention (PCI) and coronary artery bypass grafting (CABG), are considered according to symptom burden, coronary anatomy, ischemic risk, and expected prognostic benefit [1,4].

The assessment of symptom burden and quality of life (QoL) is important in CCS management. The original Seattle Angina Questionnaire (SAQ) is a validated, disease-specific instrument for assessing angina-related health status, including physical limitation, angina frequency, treatment satisfaction, and quality of life [5]. Shortened SAQ instruments have subsequently been developed to improve the feasibility of patient-reported health-status assessment in routine clinical practice [6]. In the present registry, a modified SAQ-derived questionnaire was used to capture selected aspects of physical limitation, angina burden, nitroglycerin use, and quality of life during routine outpatient follow-up. Patient-reported measures such as the SAQ provide complementary information on symptoms, functional status, and quality of life from the patient’s perspective [5,6].

The current study explored cardiologists’ real-world diagnostic and therapeutic preferences for managing CCS in outpatient settings in Turkey. In particular, the choice between medical and interventional treatment, the frequency of various diagnostic modalities, initiation patterns of new medications, and the clinical characteristics associated with medical versus interventional treatment strategies were evaluated. Further, changes in patient-reported responses to the modified SAQ-based questionnaire and clinical outcomes over the 12-week follow-up period were assessed.

## 2. Materials and Methods

This study was conducted in accordance with the ethical principles of the Declaration of Helsinki. It was approved by the Institutional Review Board and Ethics Committee of Baskent University (project No.: KA23/70; approval date: 24 May 2023). All patients provided written informed consent prior to participation in the study.

This was a prospective, multicenter observational registry study conducted in 25 cardiology outpatient clinics across Turkey between June 2023 and December 2023. In total, 762 adult patients (aged ≥18 years) evaluated in participating cardiology outpatient clinics were screened for eligibility. To focus on individuals with an established diagnosis, patients newly diagnosed with CCS (*n* = 65) were excluded from the final analysis. Ultimately, 697 patients previously diagnosed with CCS were included and prospectively followed up for 12 weeks.

### 2.1. Inclusion and Exclusion Criteria

The inclusion criteria comprised patients aged ≥18 years who had a prior diagnosis of CCS, including those with a history of stable angina, myocardial infarction, or revascularization. The exclusion criteria were patients with acute coronary syndrome, unstable angina, heart failure with reduced ejection fraction, significant valvular heart disease, active infection or inflammatory disease, malignancy, and chronic renal failure.

### 2.2. Data Collection

Demographic and clinical data were collected from the electronic medical records of the patients. The variables recorded included age, sex, body mass index, smoking status, hypertension, diabetes mellitus, hyperlipidemia, peripheral artery disease, chronic obstructive pulmonary disease, previous history of PCI or CABG, and current medications. In addition, electrocardiographic and echocardiographic findings were obtained.

The concomitant baseline cardiovascular medications of the enrolled patients, including ACEis, ARBs, beta-blockers, calcium channel blockers, nitrates, statins, and antiplatelet agents (acetylsalicylic acid, clopidogrel, ticagrelor, and prasugrel), were documented. In addition, the use of antianginal therapies such as trimetazidine, ranolazine, and ivabradine was recorded.

### 2.3. Study Groups and Outcomes

The patients were categorized into two groups based on treatment strategy:

- Medical treatment group (managed without PCI or CABG).
- Interventional treatment group (PCI or CABG, with medication changes).

The primary patient-reported outcome was the change in angina-related functional status and quality-of-life responses between baseline and 12 weeks, assessed using the modified SAQ-based questionnaire. The secondary outcomes included rates of cardiovascular hospitalization, repeat revascularization, and acute coronary syndrome (ACS) during the follow-up period. Planned staged revascularization procedures were not classified as repeat revascularization events. Similarly, procedure-related myocardial infarction or other periprocedural events related to the index intervention were not classified as follow-up ACS events.

#### SAQ

Patient-reported angina-related functional status and quality of life were assessed at baseline (T0) and at 12 weeks (T1) using a shortened, modified questionnaire derived from the Seattle Angina Questionnaire. This modified instrument was used to facilitate its practical application in routine outpatient clinical practice and consisted of seven items evaluating physical limitation during daily activities, angina frequency, nitroglycerin use, and the perceived impact of angina on quality of life. Responses were recorded using ordered categorical response options, with categories ranging from greater symptom burden or limitation to lower symptom burden or no limitation. Because the original SAQ was modified for the purposes of this registry, the conventional SAQ 0–100 domain scoring algorithm was not applied. At 12 weeks, the modified SAQ-based questionnaire was administered either during a scheduled outpatient visit or by telephone when an in-person assessment was not feasible. Patients who were hospitalized or experienced an acute coronary syndrome during follow-up remained eligible for the T1 questionnaire assessment. Based on information provided by the investigators across the 25 participating centers, there were no deaths, withdrawals, or losses to follow-up before the 12-week assessment. Consequently, paired T0–T1 questionnaire data were available for all 697 patients.

### 2.4. Statistical Analysis

Statistical analyses were performed using the Statistical Package for the Social Sciences software version 25.0 (IBM Corp., Armonk, NY, USA). According to data distribution, categorical variables were presented as the frequencies and percentages, and continuous variables were expressed as the mean ± standard deviation or median (interquartile range). Based on the normality of data assessed using the Shapiro–Wilk test, group comparisons were performed using the chi-square test for categorical variables and the independent *t*-test or the Mann–Whitney U test for continuous variables. Changes in paired categorical responses between T0 and T1 were assessed using the McNemar–Bowker test for items with more than two response categories. A *p*-value of <0.05 was considered statistically significant.

## 3. Results

### 3.1. Characteristics of the Patients

Of the 762 patients initially screened, 65 with newly diagnosed CCS were excluded from the analysis. The final cohort included 697 (91.5%) patients with an established CCS diagnosis (Table 1).

The mean age of the patients was 61.2 ± 10.1 years, and 64.0% were men. The most common comorbidities were hypertension (74.6%), hyperlipidemia (65.0%), and diabetes mellitus (42.3%). Approximately 60.0% of the patients presented with typical angina. Meanwhile, 47.1% were classified as having Canadian Cardiovascular Society class II angina and 26.4% as having class III angina. The most frequently reported angina-equivalent symptoms were dyspnea (38.0%), fatigue (25.7%), and weakness (21.9%). In terms of smoking status, 33.9% were current smokers. Meanwhile, 41.9% had quit smoking, and 24.2% had never smoked (Table 2).

### 3.2. Baseline Pharmacological Therapy

Regarding the baseline medical therapies of the study population (*N* = 697), 511 (73.3%) patients were receiving either ACEis or ARBs. Meanwhile, 527 (75.6%) patients were treated with beta-blockers. In total, 145 (20.8%) and 158 (22.7%) patients used calcium channel blockers and nitrates, respectively. Statin therapy was prescribed to 469 (67.3%) patients.

In terms of antiplatelet therapy, 591 (84.8%) patients were managed with acetylsalicylic acid, and 106 (15.2%) patients were not. Moreover, 213 (30.6%), 53 (7.6%), and 1 (0.1%) patients were treated with clopidogrel, ticagrelor, and prasugrel, respectively. Further, the prevalence of antianginal medications was reported as follows: trimetazidine, *n* = 134 patients (19.2%); ranolazine, *n* = 105 (15.1%); and ivabradine, *n* = 37 (5.3%).

To contextualize the therapeutic landscape before the 12-week observational period, the prevalence of preexisting cardiovascular and antianginal therapies was assessed. As summarized in Table 3, a substantial proportion of patients were already receiving guideline-directed cardiovascular therapies at baseline. These baseline treatment patterns are important for interpreting the subsequent rates of newly initiated medications and changes in patient-reported outcomes during follow-up.

### 3.3. Diagnostic Modalities Used

During the diagnostic evaluation, invasive coronary angiography was performed in 266 patients (38.2%), exercise treadmill testing in 220 (31.6%), myocardial perfusion imaging in 119 (17.1%), and coronary CT angiography in 39 (5.6%) (Table 4). Because more than one diagnostic modality could be performed in the same patient during the evaluation process, the diagnostic categories were not mutually exclusive.

### 3.4. Treatment Preferences

Medical therapy adjustments were more common than interventional strategies. Of the total cohort, 58.5% (*n* = 408) received medical therapy alone. Meanwhile, 41.5% (*n* = 289) underwent interventional procedures (PCI or CABG) in addition to medical therapy. Of the total cohort, 37.7% underwent PCI (*n* = 263) and 3.7% underwent CABG (*n* = 26) (Table 5).

### 3.5. Newly Initiated Medications

Among patients who were not receiving the respective medication at baseline, beta-blockers (61.8%), statins (46.5%), and ranolazine (40.4%) were the most frequently newly initiated therapies, followed by nitrates (24.3%) and trimetazidine (23.1%) (Table 6). These percentages were calculated using medication-specific denominators corresponding to patients who were not receiving each medication at baseline, rather than the total study population (Table 6).

### 3.6. Clinical Characteristics According to Treatment Strategy

When patients were compared according to the final management strategy, 289 patients were classified into the interventional treatment group (PCI or CABG, with medical therapy modification), whereas 408 patients were managed with medical therapy alone. Patients in the intervention group were significantly younger (60.4 ± 10.0 vs. 62.1 ± 10.1 years, *p* = 0.038) and were more frequently current smokers (39.1% vs. 30.1%, *p* < 0.001) than those in the medical treatment group (Table 7).

Patients in the intervention group had slightly lower LDL-C levels than those in the medical treatment group (98.8 ± 37.9 vs. 102.2 ± 39.7 mg/dL), although this difference was not statistically significant (*p* = 0.255). Weekly nitrate use was significantly higher in the intervention group (1.26 ± 2.00 vs. 0.78 ± 1.62 times per week, *p* < 0.001). Patients in the intervention group also had a significantly lower mean ejection fraction (54.1% vs. 55.9%, *p* = 0.012).

### 3.7. Results of the Modified SAQ-Based Questionnaire

Paired questionnaire data at both baseline (T0) and 12-week follow-up (T1) were available for all 697 patients included in the analysis. Patient-reported responses shifted toward less functional limitation, lower angina burden, and better perceived quality of life over the 12-week follow-up, as assessed using the modified SAQ-based questionnaire (Table 8). A clear shift toward lower levels of physical limitation was observed across daily activities. For example, the proportion of patients reporting no limitation while walking on a flat surface increased from 32.0% at baseline (T0) to 66.1% at 12 weeks (T1). Similarly, the proportion reporting no limitation in gardening increased from 18.1% to 50.4%, and that for lifting or carrying heavy objects increased from 16.4% to 43.5%. Angina burden also decreased, with the proportion of patients reporting no angina attacks during the preceding four weeks increasing from 9.5% to 45.4%. In parallel, responses regarding the impact of angina on enjoyment of life and overall satisfaction with living with angina shifted markedly toward more favorable categories at T1. Paired categorical comparisons demonstrated statistically significant changes for all items for which the McNemar–Bowker test was applicable (all *p* < 0.001) (Table 8)**.**

### 3.8. Clinical Outcomes During Follow-Up

The following findings were observed during the 12-week follow-up period:

- Approximately 5.6% of patients (*n* = 39) were hospitalized due to cardiovascular causes.
- Approximately 3.6% of the patients (*n* = 25) underwent repeat revascularization.
- Approximately 1.6% of the patients (*n* = 11) experienced acute coronary syndrome (ACS).

When stratified by treatment type, the following results were obtained:

Approximately 8.7% of patients in the intervention group and 3.4% of those in the medical group experienced cardiovascular hospitalization (*p* = 0.003). Repeat revascularization was more common in the intervention group than in the medical group (5.5% vs. 2.2%, *p* = 0.007). ACS occurred in 3.5% of patients in the intervention group versus 0.2% of those in the medical group (*p* = 0.001) (Table 9).

The higher rates of cardiovascular hospitalization, repeat revascularization, and ACS observed in the intervention group may reflect differences in baseline clinical characteristics and the non-randomized selection of patients for an interventional strategy rather than an adverse effect of the treatment strategy itself.

## 4. Discussion

This observational, multicenter study provides real-world insights into the diagnostic and therapeutic approaches of cardiologists managing patients with established CCS in Turkey. Medical therapy was the predominant treatment strategy, while exercise stress testing and invasive coronary angiography were the most frequently used diagnostic modalities. Overall, the observed patterns demonstrate the variability of diagnostic and therapeutic decision-making in routine clinical practice and should be interpreted in the context of an established CCS population and the observational design of the study.

Compared with contemporary registry-based data, our study demonstrated a different pattern of diagnostic testing in patients with established CCS. Coronary CT angiography (CCTA) was used in only 5.6% of our cohort, whereas contemporary European registry data demonstrate broader use of non-invasive anatomical and functional imaging in routine CCS assessment [7]. However, this relatively low rate should be interpreted in the context of both the study population and the period of data collection. For example, the DISCHARGE trial evaluated a CCTA-based diagnostic strategy in patients with stable chest pain referred for invasive coronary angiography, a population distinct from the exclusively established CCS cohort evaluated in the present study [8]. Unlike these populations, all patients included in the present analysis had previously established CCS, including patients with prior myocardial infarction or coronary revascularization. Furthermore, patient enrollment was conducted between June and December 2023, before publication of the 2024 ESC Guidelines for the management of CCS. Therefore, the observed CCTA utilization rate should not be interpreted as evidence of non-adherence to the subsequent 2024 recommendations. In patients with established CAD, the selection of diagnostic testing may also be influenced by previous coronary anatomy or revascularization, clinical characteristics, local availability, institutional workflow, and expertise with individual imaging modalities. Exercise stress testing and invasive coronary angiography were used more frequently in our cohort, in 31.6% and 38.2% of patients, respectively. Accordingly, the relatively low use of CCTA in this registry should primarily be regarded as a descriptive finding reflecting real-world diagnostic practice in patients with established CCS during the study period rather than as evidence of inappropriate underutilization [1].

Regarding pharmacological therapy, among patients who were not receiving the respective medication at baseline, beta-blockers (61.8%), statins (46.5%), and ranolazine (40.4%) were the most frequently newly initiated therapies. These findings should be interpreted in the context of the high baseline use of guideline-directed medical therapies, particularly beta-blockers (75.6%) and statins (67.3%). High utilization of secondary-prevention therapies has also been observed in large international CCS registries. In CLARIFY, for example, beta-blockers and statins were used in approximately 75% and 83% of patients, respectively [9]. Because treatment adherence, dose intensity, and achievement of therapeutic targets were not evaluated, the present data do not allow conclusions regarding the overall adequacy of guideline-directed pharmacological therapy.

Regarding therapeutic choices, the ISCHEMIA trial showed that an initial invasive strategy did not reduce the risk of ischemic cardiovascular events or death compared with an initial conservative strategy in patients with stable coronary disease and moderate or severe ischemia [10]. However, the invasive strategy provided greater improvement in angina-related symptoms and quality of life, particularly among patients with more frequent angina at baseline [11]. In addition, the relatively high proportion of patients who received medical treatment (58.5%) is consistent with the central role of guideline-directed medical therapy in the management of CCS. The COURAGE trial demonstrated that, in patients with stable coronary disease, the addition of PCI to optimal medical therapy did not reduce the risk of death or myocardial infarction compared with optimal medical therapy alone [12]. Nevertheless, PCI provided greater improvement in angina-related health status during the early follow-up period, although this incremental benefit diminished over longer-term follow-up [13].

Patients in the intervention group were younger, more likely to be current smokers, had greater weekly nitrate use, and had lower ejection fraction than those managed medically. These between-group differences may have influenced treatment selection; however, multivariable logistic regression was not performed to determine whether these factors independently predicted the selection of an invasive strategy. This is considered a limitation of the study and should be addressed in future analyses to better understand treatment decision-making.

Patient-reported responses to the modified SAQ-based questionnaire shifted toward less functional limitation, lower angina burden, and better perceived quality of life over the 12-week follow-up. Importantly, because a modified SAQ-derived instrument rather than the conventional SAQ scoring algorithm was used, the observed changes represent changes in individual patient-reported questionnaire items rather than changes in validated SAQ domain scores. Therefore, these findings should be interpreted as changes in patient-reported angina-related health status rather than changes in conventional SAQ scores. Longer-term studies using standardized patient-reported outcome measures are warranted to confirm these findings.

The improvement in patient-reported angina-related health status observed during follow-up is broadly consistent with evidence that contemporary management of CCS can improve symptom burden and quality of life. These observations are also consistent with contemporary real-world data. In the EORP EURECA registry, improvement in anginal symptoms and quality of life was observed at 6 months, with greater improvement among patients undergoing revascularization than among those managed by medical-treatment modification or without treatment change [14]. In the ISCHEMIA quality-of-life substudy, patients with more frequent angina at baseline who were initially managed with an invasive strategy experienced greater improvement in validated SAQ summary scores than those treated conservatively [15]. Direct comparison with our findings is not appropriate because our study used a modified SAQ-derived questionnaire without the conventional scoring algorithm. In addition, differences in baseline symptom burden, follow-up duration, and treatment allocation should be considered when interpreting these findings [11,13].

Cardiovascular events such as hospitalization, repeat revascularization, and acute coronary syndrome were more frequently observed in the intervention group than in the medical group. This may reflect differences in baseline clinical risk and complexity between the treatment groups rather than an adverse effect of the interventional strategy itself. Nonetheless, these results emphasize the need for cautious risk stratification and individualized treatment planning.

### Study Limitations

This study has several limitations. First, its observational design precludes causal inference between treatment strategies and clinical outcomes. Second, the relatively short follow-up period (12 weeks) limits the assessment of longer-term cardiovascular events and sustained changes in symptoms and quality of life. Contemporary CCS registries have assessed these outcomes over longer follow-up intervals, including at 6 months [14]. Third, treatment allocation was not randomized, and residual confounding is likely to exist. Finally, the absence of detailed risk stratification models limits a deeper evaluation of decision-making processes in invasive versus conservative management.

An additional limitation is that the registry used a modified questionnaire derived from the SAQ rather than the original validated SAQ scoring system; therefore, the results cannot be directly compared with conventional SAQ domain or summary scores reported in other studies.

## 5. Conclusions

In this real-world, multicenter registry of patients with established CCS in Turkey, medical therapy was the predominant treatment approach, while a substantial proportion of patients were managed with an interventional strategy. Diagnostic evaluation showed considerable use of exercise stress testing and invasive coronary angiography, whereas CCTA was used less frequently. Baseline use of guideline-directed pharmacological therapies, particularly beta-blockers and statins, was high, and additional therapies were initiated according to individual treatment requirements. These findings provide a descriptive overview of contemporary outpatient CCS management in Turkey and should be interpreted in the context of the observational study design and the established CCS population.

Future studies with longer follow-up and multivariable analyses are warranted to better identify determinants of treatment selection and clinical outcomes. More detailed assessment of medication adherence, treatment intensity, achievement of therapeutic targets, and the factors influencing the selection of noninvasive versus invasive diagnostic strategies may further clarify opportunities to optimize CCS management.

## Figures and Tables

**Table 1:** Distribution of patients according to history of coronary artery disease.

Coronary Artery Disease Status	* n *	%
Previous diagnosis of CAD	697	91.5%
New diagnosis of CAD (excluded)	65	8.5%
Total number of patients evaluated	762	100%

**Table 2:** Demographic and clinical characteristics of the study population.

Variables	Mean ± SD/*N* (%)
Age (years)	61.22 ± 10.06
Sex (male)	446 (64.0%)
Typical angina	418 (60.0%)
Atypical angina	258 (37.0%)
Non-angina chest pain	18 (2.6%)
Absence of chest pain	3 (0.4%)
CCS-AC class I	163 (23.4%)
CCS-AC class II	328 (47.1%)
CCS-AC class III	184 (26.4%)
CCS-AC class IV	22 (3.2%)
Dyspnea	265 (38.0%)
Fatigue	179 (25.7%)
Weakness	153 (21.9%)
Nausea	33 (4.7%)
Sweating	110 (15.8%)
Absence of angina-equivalent symptoms	185 (26.5%)
Hypertension	520 (74.6%)
Diabetes mellitus	295 (42.3%)
Hyperlipidemia	453 (65.0%)
Peripheral artery disease	40 (5.7%)
COPD	75 (10.8%)
Smoking (current)	236 (33.9%)
Smoking (former)	292 (41.9%)
Never smoked	169 (24.2%)

CCS-AC, Canadian Cardiovascular Society angina class.

**Table 3:** Distribution of baseline medications used by the participants.

Medications/Variables	Status	*n* (%)
ACE inhibitors	Yes	348 (49.9)
	No	349 (50.1)
ARB	Yes	163 (23.4)
	No	534 (76.6)
Beta-blockers	Yes	527 (75.6)
	No	170 (24.4)
Calcium channel blockers	Yes	145 (20.8)
	No	552 (79.2)
Nitrates	Yes	158 (22.7)
	No	539 (77.3)
Statins	Yes	469 (67.3)
	No	228 (32.7)
ASA	Yes	591 (84.8)
	No	106 (15.2)
Clopidogrel	Yes	213 (30.6)
	No	484 (69.4)
Ticagrelor	Yes	53 (7.6)
	No	644 (92.4)
Prasugrel	Yes	1 (0.1)
	No	696 (99.9)
Trimetazidine	Yes	134 (19.2)
	No	563 (80.8)
Ranolazine	Yes	105 (15.1)
	No	592 (84.9)
Ivabradine	Yes	37 (5.3)
	No	660 (94.7)
Total		697 (100.0)

Abbreviations: ACE, angiotensin-converting enzyme; ARB, angiotensin receptor blocker; ASA, acetylsalicylic acid.

**Table 4:** Diagnostic modalities used during the evaluation of the study population.

Diagnostic Modality	* n *	%
Exercise stress test	220	31.6%
Myocardial perfusion imaging	119	17.1%
Dobutamine stress echocardiogram	1	0.1%
Invasive coronary angiography	266	38.2%
Coronary CT angiography	39	5.6%
None	59	8.5%

More than one diagnostic modality could be performed in the same patient; therefore, the categories were not mutually exclusive and the total number of diagnostic procedures may exceed the number of patients. Percentages were calculated using the total study population (*N* = 697) as the denominator.

**Table 5:** Patient management after evaluation.

Treatment Strategy	* n *	%
Medical therapy alone	408	58.5%
Interventional + medical therapy	289	41.5%

**Table 6:** Newly initiated medications among patients not receiving the corresponding medication at baseline.

Medications	* n *	%
Ranolazine	239	40.4%
Nitrates	131	24.3%
Trimetazidine	130	23.1%
Statins	106	46.5%
Beta-blockers	105	61.8%

Percentages were calculated using medication-specific denominators, defined as the number of patients who were not receiving the corresponding medication at baseline and were therefore eligible for new initiation, rather than the total study population (*N* = 697). Baseline medication use and the corresponding medication-specific denominators are presented in Table 3.

**Table 7:** Clinical characteristics of the treatment groups.

Parameters	Medical Treatment Group (*n* = 408)	Intervention Group (*n* = 289)	*p*-Value
Age (years)	62.05 ± 10.12	60.37 ± 9.98	0.038 *
Male sex	63.5%	64.7%	0.740
Current smoking	30.1%	39.1%	<0.001 *
LDL-C level (mg/dL)	102.22 ± 39.69	98.81 ± 37.90	0.255
Stress test duration (min)	3.96 ± 2.55	4.73 ± 3.54	0.077
Nitrate use (per week)	0.78 ± 1.62	1.26 ± 2.00	<0.001 *
Ejection fraction (%)	55.89 ± 7.34	54.06 ± 8.92	0.012 *

* Patients in the intervention group were significantly younger, were more frequently current smokers, used nitrates more frequently each week, and had lower EF values.

**Table 8:** Changes in responses to the modified SAQ-based questionnaire between baseline and 12-week follow-up.

Questionnaire Item	Response Category	T0, *n* (%)	T1, *n* (%)	McNemar–Bowker Test, χ^2^ (*p*)
Walking on a flat surface at home	Extremely limited	9 (1.3)	0 (0.0)	—
	Very limited	53 (7.6)	7 (1.0)	
	Moderately limited	107 (15.4)	36 (5.2)	
	Slightly limited	305 (43.8)	193 (27.7)	
	Not limited at all	223 (32.0)	460 (66.1)	
Gardening	Extremely limited	19 (2.7)	1 (0.1)	345.670 (*p* < 0.001)
	Very limited	90 (12.9)	12 (1.7)	
	Moderately limited	161 (23.1)	60 (8.6)	
	Slightly limited	301 (43.2)	272 (39.1)	
	Not limited at all	126 (18.1)	351 (50.4)	
Lifting or carrying heavy objects, or carrying a child	Extremely limited	37 (5.3)	1 (0.1)	348.346 (*p* < 0.001)
	Very limited	86 (12.3)	15 (2.2)	
	Moderately limited	181 (26.0)	57 (8.2)	
	Slightly limited	279 (40.0)	320 (46.0)	
	Not limited at all	114 (16.4)	303 (43.5)	
How often have you experienced angina attacks during the past 4 weeks?	≥4 times/day	30 (4.3)	2 (0.3)	418.581 (*p* < 0.001)
	1–3 times/day	104 (14.9)	17 (2.4)	
	≥3 times/week, but not every day	211 (30.3)	74 (10.6)	
	1–2 times/week	286 (41.0)	287 (41.2)	
	None	66 (9.5)	316 (45.4)	
How often have you used nitroglycerin (NTG) during the past 4 weeks?	≥4 times/day	10 (1.4)	0 (0.0)	—
	1–3 times/day	39 (5.6)	15 (2.2)	
	≥3 times/week, but not every day	82 (11.8)	28 (4.0)	
	1–2 times/week	114 (16.4)	162 (23.3)	
	None	452 (64.8)	491 (70.5)	
To what extent has angina limited your enjoyment of life?	Extremely limited	52 (7.5)	3 (0.4)	414.449 (*p* < 0.001)
	Very limited	149 (21.4)	18 (2.6)	
	Moderately limited	180 (25.8)	77 (11.1)	
	Slightly limited	271 (38.9)	262 (37.6)	
	Not limited at all	45 (6.5)	336 (48.3)	
If you had to spend the rest of your life with your angina attacks as they are now, how would you feel?	I would not be satisfied at all	291 (41.8)	80 (11.5)	398.106 (*p* < 0.001)
	I would not be satisfied	368 (52.8)	216 (31.0)	
	I would be very satisfied	38 (5.5)	400 (57.5)	

Data are presented as *n* (%). T0, baseline; T1, 12-week follow-up; NTG, nitroglycerin. Paired T0 and T1 questionnaire data were available for all 697 patients. McNemar–Bowker test was used for paired categorical comparisons where applicable. McNemar–Bowker statistics were not reported for the walking-on-a-flat-surface and nitroglycerin-use items because sparse and zero cells in the paired contingency tables precluded reliable asymptotic estimation. Statistical significance was defined as *p* < 0.05.

**Table 9:** Cardiovascular events during follow-up.

Clinical Events	Medical Treatment Group (*n* = 408)	Intervention Group (*n* = 289)	*p*-Value
CV-related hospitalization	14 (3.4%)	25 (8.7%)	0.003 *
Repeat revascularization	9 (2.2%)	16 (5.5%)	0.007 *
Acute coronary syndrome	1 (0.2%)	10 (3.5%)	0.001 *

* Cardiovascular-related hospitalization, repeat revascularization, and acute coronary syndrome were significantly less frequent in the medical treatment group than in the intervention group. Planned staged revascularization procedures were not counted as repeat revascularization events, and procedure-related myocardial infarction or other periprocedural events related to the index intervention were not counted as follow-up ACS events.

## Data Availability

The research data may be shared upon request by the editor of the journal.

## Abbreviations

The following abbreviations are used in this manuscript:

CAD	Coronary Artery Disease
ACE	Angiotensin Converting Enzyme
ARB	Angiotensin Receptor Blocker
CCS	Chronic Coronary Syndrome
COPD	Chronic Obstructive Pulmonary Disease
ASA	Acetylsalicylic acid
SAQ	Seattle Angina Questionnaire
QoL	Quality of Life
CCS-AC	Canadian Cardiovascular Society angina class

## References

[1] Vrints C., Andreotti F., Koskinas K.C., Rossello X., Adamo M., Ainslie J., Banning A.P., Budaj A., Buechel R.R., Chiariello G.A. (2024). 2024 ESC Guidelines for the management of chronic coronary syndromes. Eur. Heart J..

[2] Safiri S., Karamzad N., Singh K., Carson-Chahhoud K., Adams C., Nejadghaderi S.A., Almasi-Hashiani A., Sullman M.J.M., Mansournia M.A., Bragazzi N.L. (2022). Burden of ischemic heart disease and its attributable risk factors in 204 countries and territories, 1990–2019. Eur. J. Prev. Cardiol..

[3] Yalım Z., Doğan N., Alan Yalım S. (2022). Mortality trends from ischemic heart disease in Turkey: 2009–2019. Turk Kardiyol. Dern. Ars..

[4] Virani S.S., Newby L.K., Arnold S.V., Bittner V., Brewer L.C., Demeter S.H., Dixon D.L., Fearon W.F., Hess B., Johnson H.M. (2023). 2023 AHA/ACC/ACCP/ASPC/NLA/PCNA Guideline for the Management of Patients With Chronic Coronary Disease: A Report of the American Heart Association/American College of Cardiology Joint Committee on Clinical Practice Guidelines. Circulation.

[5] Spertus J.A., Winder J.A., Dewhurst T.A., Deyo R.A., Prodzinski J., McDonell M., Fihn S.D. (1995). Development and evaluation of the Seattle Angina Questionnaire: A new functional status measure for coronary artery disease. J. Am. Coll. Cardiol..

[6] Chan P.S., Jones P.G., Arnold S.A., Spertus J.A. (2014). Development and validation of a short version of the Seattle Angina Questionnaire. Circ. Cardiovasc. Qual. Outcomes.

[7] Neglia D., Liga R., Gimelli A., Podlesnikar T., Cvijić M., Pontone G., Miglioranza M.H., Guaricci A.I., Seitun S., Clemente A. (2023). Use of cardiac imaging in chronic coronary syndromes: The EURECA Imaging registry. Eur. Heart J..

[8] DISCHARGE Trial Group (2022). CT or Invasive Coronary Angiography in Stable Chest Pain. N. Engl. J. Med..

[9] Sorbets E., Fox K.M., Elbez Y., Danchin N., Dorian P., Ferrari R., Ford I., Greenlaw N., Kalra P.R., Parma Z. (2020). Long-term outcomes of chronic coronary syndrome worldwide: Insights from the international CLARIFY registry. Eur. Heart J..

[10] Maron D.J., Hochman J.S., Reynolds H.R., Bangalore S., O’Brien S.M., Boden W.E., Chaitman B.R., Senior R., López-Sendón J., Alexander K.P. (2020). Initial invasive or conservative strategy for stable coronary disease. N. Engl. J. Med..

[11] Spertus J.A., Jones P.G., Maron D.J., O’Brien S.M., Reynolds H.R., Rosenberg Y., Stone G.W., Harrell F.E., Boden W.E., Weintraub W.S. (2020). Health-status outcomes with invasive or conservative care in coronary disease. N. Engl. J. Med..

[12] Boden W.E., O’Rourke R.A., Teo K.K., Hartigan P.M., Maron D.J., Kostuk W.J., Knudtson M., Dada M., Casperson P., Harris C.L. (2007). Optimal medical therapy with or without PCI for stable coronary disease. N. Engl. J. Med..

[13] Weintraub W.S., Spertus J.A., Kolm P., Maron D.J., Zhang Z., Jurkovitz C., Zhang W., Hartigan P.M., Lewis C., Veledar E. (2008). Effect of PCI on quality of life in patients with stable coronary disease. N. Engl. J. Med..

[14] Liga R., Gimelli A., Podlesnikar T., Cvijić M., Pontone G., Miglioranza M.H., Guaricci A.I., Seitun S., Clemente A., Sumin A. (2025). Treatment and outcomes of chronic coronary syndromes: The EORP EURECA imaging registry. Eur. Heart J..

[15] Mark D.B., Spertus J.A., Bigelow R., Anderson S., Daniels M.R., Anstrom K.J., Baloch K.N., Cohen D.J., Held C., Goodman S.G. (2022). Comprehensive quality-of-life outcomes with invasive versus conservative management of chronic coronary disease in ISCHEMIA. Circulation.

